# Venoarterial extracorporeal membrane oxygenation in elective high-risk percutaneous coronary intervention: a viable option?

**DOI:** 10.1007/s12471-020-01372-7

**Published:** 2020-02-10

**Authors:** C. L. Meuwese, F. Z. Ramjankhan, A. O. Kraaijeveld, D. W. Donker

**Affiliations:** 1 Department of Intensive Care, University Medical Centre Utrecht, Utrecht, The Netherlands; 2 Department of Cardiothoracic Surgery, University Medical Centre Utrecht, Utrecht, The Netherlands; 3 Department of Cardiology, University Medical Centre Utrecht, Utrecht, The Netherlands

Venoarterial extracorporeal membrane oxygenation (ECMO) has become a mainstay therapy for patients with severe cardiogenic shock [1]. Elective deployment of ECMO as prophylactic circulatory support to avoid refractory cardiac arrest or development of shock during high-risk percutaneous coronary interventions (PCI) has not yet unequivocally been accepted as procedural routine in the catheterisation laboratory.

In the current edition of the *Netherlands Heart Journal*, van den Brink et al. [2] describe a cohort of 14 patients in whom peripheral ECMO was used to ensure adequate systemic perfusion during high-risk elective PCI. The intervention was successful in 100% of cases and 13 out of 14 subjects (93%) survived until hospital discharge. Yet, complications occurred in 2 out of 24 patients (14%) with transient ischaemic attack and femoral thrombosis both taking place in 1 patient each.

In the absence of randomised trials, causal effects of ECMO for prevention of shock and mortality in elective, high-risk PCI remain unknown. Empirically and mechanistically, ECMO has been recognised as a powerful tool to safeguard tissue perfusion in the face of severe cardiac dysfunction. Application of ECMO nevertheless can take its toll through a diversity of severe complications, including those related to vascular access and to the extracorporeal circuit as such. Vascular dissection or perforation (both arterial and venous), thromboembolism and significant bleeding, limb ischaemia, and ischaemic stroke are well-described and relatively common complications of venoarterial ECMO. Particularly in PCI patients in whom anticoagulation and antithrombotic agents are often combined, the elective use of ECMO requires careful weighing-up and planning by an experienced multidisciplinary team [1].

With these potential risks in mind, three questions can be asked:

1. Does the advantage of revascularisation in terms of mortality benefit and/or symptom reduction outweigh its potential risks and additional costs?
2. Which patients are at highest risk for haemodynamic compromise during the procedure and may benefit from prophylactic venoarterial ECMO support?
3. Which conditions might mitigate the significant risks imposed by ECMO and may other temporary circulatory support devices, e.g. microaxial balloon pump (Impella), represent a safer strategy?

With respect to the first question, it is important to highlight the fact that patients with poor left ventricular function seem to profit most from revascularisation when their survival expectation exceeds 4 years [3, 4]. This computation could become negatively affected by the increased risk involved with application of ECMO (or the increase in risk represented by ECMO for a specific procedure), the extent of which cannot yet be established because of the scarcity of data. For patients whose long-term prognosis is overshadowed by other comorbidities, this could serve as an argument to refrain from revascularisation. The current study highlights the fundamental role of the heart team in this difficult decision-making process.

Regarding the second aspect, it is not known in which patients the procedural risk may be alleviated by prophylactic ECMO application. American, but not European, guidelines suggest considering prophylactic ECMO in high-risk elective PCI procedures, defined as the presence of unprotected left main stenosis, PCI of the last remaining conduit, PCI of a vessel that covers a large territory on a background of poor left ventricular function, or cardiogenic shock [5]. For further specification of hazard, several prediction formulas (e.g. EuroSCORE and SYNTAX) aid in the calculation of the short-term mortality risk [2]. Extrapolating these results to assess whether prophylactic ECMO support is indicated may, however, be troublesome, as this assumption demands that causal pathways leading to death can directly be influenced and averted by the prophylactic use of ECMO. This postulation might, however, be too shortsighted, as many adverse postprocedural events such as cardiac arrest, in-stent thrombosis, bleeding and infection can occur independently of haemodynamic compromise during a procedure and are in turn associated with deterioration and death [6]. To our knowledge, no studies have examined the predictive value of one of these algorithms for the probability of haemodynamic collapse during an intervention. Alternatively, it has been postulated that left ventricular filling pressures would predict the chance of haemodynamic compromise during a procedure. No association, however, was found between left ventricular end-diastolic pressure and the occurrence of haemodynamic collapse in a study examining the safety profile of Impella during elective high-risk PCI procedures [7].

Finally, it seems important to optimise conditions in order to mitigate the risks imposed by ECMO. After the initial experience with elective, surgically cannulated peripheral ECMO in high-risk PCI in the Netherlands more than 10 years ago [8], percutaneous access, preferably in conjunction with a distal selective perfusion cannula, has become a gold standard for vascular ECMO access. Of interest in the current study is that all patients were intubated preemptively. Although ECMO can entirely be performed without mechanical ventilation [9], mechanical ventilation may provide beneficial effects, especially when procedures become complicated and prolonged. These may include the possibility for adequate analgosedation and delivery of positive end-expiratory pressure, which could prevent the development of overt pulmonary oedema. If high ECMO flow rates would inevitably result in left ventricular overload, functional and structural myocardial compromise and pulmonary oedema (and the related risk of the so-called Harlequin syndrome), left ventricular unloading strategies may be warranted [1, 10].

The study by van den Brink et al. [2] lays an important piece of a complicated puzzle, thereby contributing to a growing body of evidence that ECMO can be successfully applied as prophylactic support in patients undergoing high-risk PCI procedures. There is a need for additional studies to further (1) quantify the risks and advantages of high-risk PCI procedures under prophylactic ECMO support, (2) identify predictors for haemodynamic collapse during high-risk PCI procedures to stratify specific patients who may in particular benefit from this strategy, and (3) determine optimal circumstances to mitigate the risks involved with ECMO and elaborate on alternative short-term mechanical support strategies, for instance the Impella system.
